# Al_2_Pt for Oxygen Evolution in Water Splitting: A Strategy for Creating Multifunctionality in Electrocatalysis

**DOI:** 10.1002/anie.202005445

**Published:** 2020-07-10

**Authors:** Iryna Antonyshyn, Ana M. Barrios Jiménez, Olga Sichevych, Ulrich Burkhardt, Igor Veremchuk, Marcus Schmidt, Alim Ormeci, Ioannis Spanos, Andrey Tarasov, Detre Teschner, Gerardo Algara‐Siller, Robert Schlögl, Yuri Grin

**Affiliations:** ^1^ Max-Planck-Institut für Chemische Physik fester Stoffe Nöthnitzer Str. 40 01187 Dresden Germany; ^2^ Max-Planck-Institut für Chemische Energiekonversion Stiftstraße 34–36 45470 Mülheim an der Ruhr Germany; ^3^ Fritz-Haber-Institut der Max-Planck-Gesellschaft Faradayweg 4–6 14195 Berlin Germany

**Keywords:** electrocatalysis, intermetallic phases, water splitting

## Abstract

The production of hydrogen via water electrolysis is feasible only if effective and stable catalysts for the oxygen evolution reaction (OER) are available. Intermetallic compounds with well‐defined crystal and electronic structures as well as particular chemical bonding features are suggested here to act as precursors for new composite materials with attractive catalytic properties. Al_2_Pt combines a characteristic inorganic crystal structure (anti‐fluorite type) and a strongly polar chemical bonding with the advantage of elemental platinum in terms of stability against dissolution under OER conditions. We describe here the unforeseen performance of a surface nanocomposite architecture resulting from the self‐organized transformation of the bulk intermetallic precursor Al_2_Pt in OER.

## Graphical abstract

**Unforeseen performance**: The study of the chemical and morphological transformation of intermetallic Al_2_Pt, a precursor of a new composite material, under the harsh oxidative conditions of the oxygen evolution reaction (OER) is accompanied by its electrochemical characterization at different stages of long‐term operation.

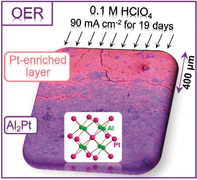

## Introduction

The global demand for transportable renewable electricity positions hydrogen as the universal first product of chemical energy conversion.^1^, ^2^, ^3^, ^4^, ^5^, ^6^, ^7^ For the production of hydrogen via electrolysis, the technique of choice needs to be scalable to global dimensions, posing challenges for the development of oxygen evolution reaction (OER) catalyst materials capable of operating under the dynamic load provided by renewable electricity.^8^, ^9^, ^10^ Proton‐exchange membrane (PEM) electrolysis has a variety of advantages (operation at high current densities, low gas crossover, compact system design etc.) compared to the well‐established alkaline variant.^8^ One of the primary challenges for the further development of PEM electrolysis is minimizing the use of iridium compounds as anode materials. Despite the increasing interest in the earth‐abundant catalysts based on manganese oxides,^11^, ^12^, ^13^, ^14^, ^15^, ^16^, ^17^, ^18^ the number of effective catalysts in acidic media are mainly restricted to noble metals and their oxides.^19^, ^20^, ^21^ The basic reason for this is the electrode stability issue under the harsh oxidation conditions of OER (evolution of reactive forms of oxygen, presence of acidic electrolyte, and applied potential). Therefore, the search for an alternative catalyst to Ir, combining activity and stability, is a key demand.

There are two basic strategies for the improvement of electrocatalyst activity: 1) increasing the number of active sites (e.g., via nanostructuring, shaping or using supports) and 2) increasing the intrinsic activity of electrocatalyst (e.g., alloying, synthesis of core–shell catalysts etc.).^22^ The opportunities to tailor the intrinsic activity of a catalyst are conceptually unlimited by optimizing the local electronic structure.^23^ Alloying is a common methodology for shaping the electronic structure; however, it has the disadvantages of disordered local structures and of a complex segregation behavior. In contrast to metallic solid solutions, intermetallic compounds with their well‐defined crystal structure and chemical bonding situation provide a platform for fundamental catalytic studies.^24^, ^25^, ^26^ In this work, we describe a novel material development strategy that combine the conflicting goals of high activity in OER and high stability under harsh oxidative conditions. For this, we use the concept of reducing the density of states at the Fermi level of Pt by combining it with a main group element that introduces strong covalency into the chemical bonding, thereby increasing chemical stability—in particular—against oxidation. This is the key idea to prevent continuous segregation and decomposition of the intermetallic material. Compensation for the resulting loss in specific activity is sought by using the self‐controlled dissolution of the main group element, creating a roughened or porous surface structure on an initially low‐surface‐area metallic material. The self‐control should be achieved through an amphoteric character of the dissolution product under the experimental conditions, leading to a soft passivation layer that confines the dissolution process to the near‐surface region and makes it possible to use the intermetallic compound as a current collector without having to create an additional chemically sensitive interface between the active material and its back‐contact that is exposed to the electrolyte.

Elemental platinum was principally ignored as a catalyst of choice for the OER since it has one of the highest overpotentials (730 mV at current density of 5 mA cm^−2^) compared to other noble metals (e.g. 370 mV for Ir and 220 mV for Ru). Conversely, Pt was found to be a highly stable catalyst with the lowest dissolution rate among the noble metals.^20^, ^27^, ^28^ Replacing elemental platinum by a Pt‐containing intermetallic compound opens a new route to reducing the OER overpotential while maintaining the desired stability. The choice of Al_2_Pt among a variety of Pt‐based intermetallic compounds was done based on pronounced covalent atomic interactions in Al_2_Pt, supported by significant charge transfer from Al to Pt,^29^ which may have influence on the electrochemical behavior.

## Results and Discussion

**Figure 1:**
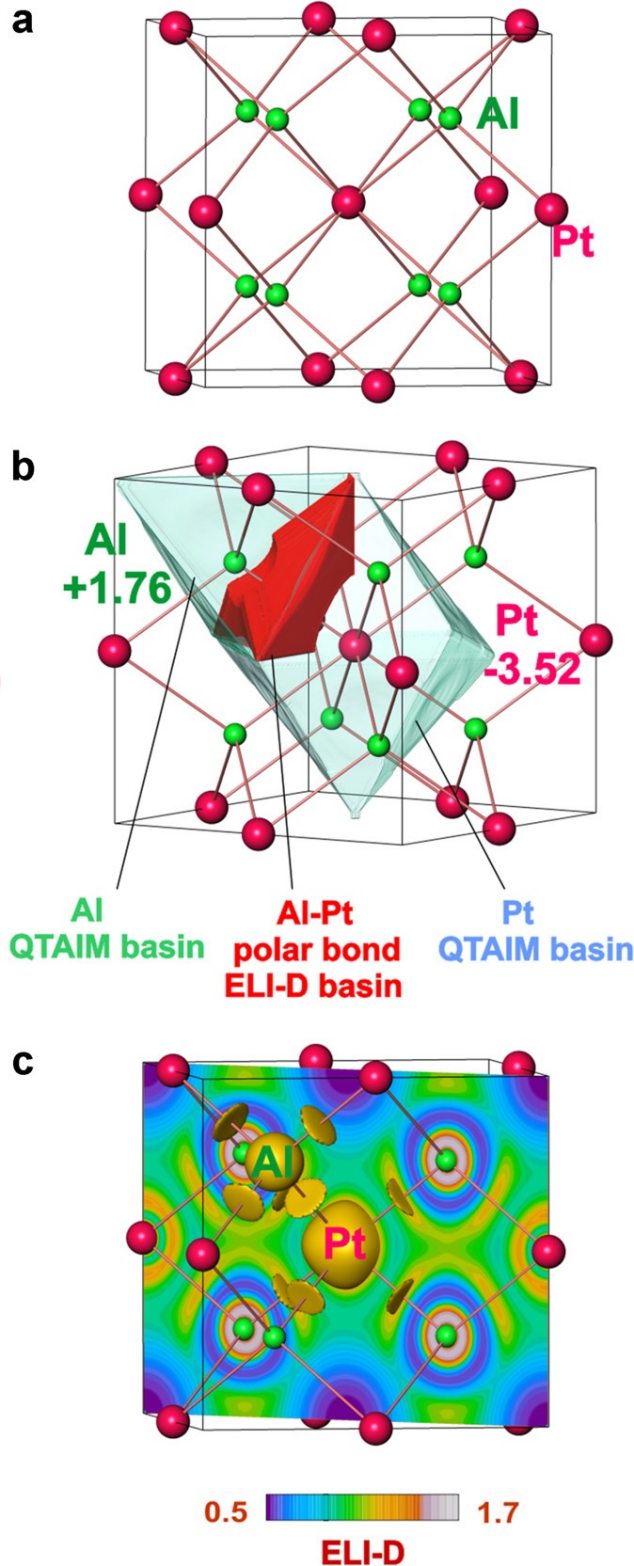
Crystal structure and chemical bonding in Al_2_Pt: a) crystal structure with the shortest Al–Pt distances; b) atomic QTAIM basins of platinum and aluminum (transparent) and the Al–Pt bond basin (red); c) distribution of ELI‐D in a ($1\bar{1}0$ )) plane and isosurface with ELI‐D=1.13, visualizing the Al–Pt bonding in the position space.

The intermetallic compound Al_2_Pt crystallizes with the cubic structure of anti‐fluorite (anti‐CaF_2_ or Na_2_O) type.^30^, ^31^, ^32^, ^33^ In this structure, aluminum atoms are located on the fluorine positions in the prototype, and platinum atoms occupy the calcium sites. Each platinum is surrounded by eight aluminum atoms forming a cube; each aluminum has four Pt atoms as ligands forming a tetrahedron. Eight four‐bonded Al atoms and four eight‐bonded Pt atoms form a 3D framework (Figure 1 a). The quantum chemical calculations reveal platinum atoms with negative QTAIM (quantum theory of atoms in molecules) charges (−3.52), which are in fair agreement with earlier published results.^29^ A charge transfer of 1.76 electrons takes place from each aluminum towards platinum atoms (Figure 1 b). Position‐space analysis of the chemical bonding applying the electron localizability indicator (ELI, Figure 1 c) reveals only one type of Al–Pt interactions with 1.14 electrons per bond. This interaction is strongly polar: 0.84 electrons are contributed by platinum compared to only 0.30 originating from the aluminum ligand, as obtained from the intersection analysis of the atomic QTAIM and ELI‐D bond basins. From the point of view of chemical bonding, the platinum species in Al_2_Pt are not equivalent to Pt in element. It is thus not a surprise that the OER overpotential for the pristine Al_2_Pt drops substantially down to 580 mV for delivering a current density of 10 mA cm^−2^.

**Figure 2:**
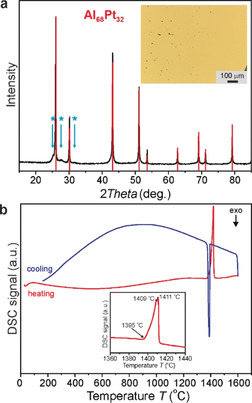
As‐synthesized Al_2_Pt: a) powder X‐ray diffraction pattern of the Al_68_Pt_32_ sample (red lines: positions and relative intensities of the Al_2_Pt phase, blue arrows: peaks caused possibly by the modulation of the crystal structure); inset: single‐phase microstructure (light microscopy; bright field contrast). b) Thermal behavior under Ar (inset: temperature region of the peritectic reaction).

The single‐phase material for electrochemical studies was synthesized using nominal composition Al_68_Pt_32_ with the arc melting technique (for details, see the Supporting Information, SI). The goldish metallic, brittle specimen was characterized by bulk‐sensitive techniques (Figure 2). The X‐ray powder diffraction pattern is indexed using the cubic‐face‐centered unit cell with a lattice parameter *a=*5.9190(2) Å (Figure 2 a). Metallographic analysis of the Al_68_Pt_32_ sample excludes any secondary phases in the synthesized material as well as any other elements, except the constituent Al and Pt (inset of Figure 2 a and Figure S1). Based on a wavelength‐dispersive X‐ray spectroscopy (WDXS) study, the molar ratio Al/Pt=68.07(2):31.93(2) is found to be close to the nominal composition. Differential scanning calorimetry (DSC) confirmed the phase purity (Figure 2 b): one signal was detected with an onset temperature of 1395 °C, which is close to the reported temperatures of 1400–1406 °C for the peritectic reaction Al_2_Pt→Al_3_Pt_2_ + *L*_._ 33 The broad DSC signal represents both the peritectic reaction and the liquidus, with very close temperatures, which is in agreement with the Al–Pt phase diagram.^33^

**Figure 3:**
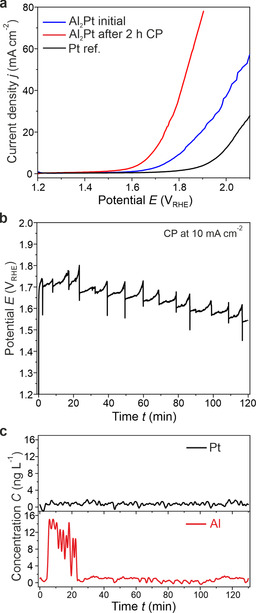
Electrochemical behavior of Al_2_Pt in 0.1 m HClO_4_: a) linear sweep voltammetry of as‐prepared material (blue) and after 2 h at 10 mA cm^−2^ (red) compared to a Pt reference (black); b) chronopotentiometry for 2 h at 10 mA cm^−2^, accompanied by c) elemental analysis of effluent electrolyte.

Prior to electrochemical activity measurements, cyclic voltammetry (CV) was performed on the platinum and the Al_2_Pt catalysts to free the surface from possible organic contaminants as well as to inspect the Pt redox features (Figure S2). The electrochemical activity of Al_2_Pt towards OER was estimated based on linear sweep voltammetry (LSV) performed up to 2.1 V_RHE_. The OER activities were judged by the overpotential values at a current density of 10 mA cm^−2^, which is the benchmarking value for comparison of OER electrocatalysts at the stage of fundamental studies.^34^, ^35^ The comparison of such overpotentials for Al_2_Pt and elemental Pt anode materials reveals a difference of 170 mV in favor of Al_2_Pt (Figure 3 a). After 2 h of the chronopotentiometry (CP) experiment at 10 mA cm^−2^ (Figure 3 b), at least two remarkable changes were observed: 1) the slope of the LSV curve changes strongly due to the improved mass transport after removal of a likely passivation layer of alumina (Figure 3 a);^36^, ^37^, ^38^, ^39^ and 2) the OER overpotential shifts towards lower potential values (450 mV instead of initial 580 mV at 10 mA cm^−2^, Figure 3 a, cf. 750 mV for the elemental Pt), revealing ongoing changes of the catalyst material (Figure 3 b). Despite the notable current densities, which may be affected by changes in the electrochemically active surface area after removal of surface alumina, the onset potential of oxygen evolution is still about 18 % lower for the Al_2_Pt after 2 h CP compared to *fcc* Pt (Figure 3 a). According to the elemental analysis of effluent electrolyte, no platinum dissolution (<0.05 mg L^−1^) and only a small amount of dissolved Al during the first few minutes of the stress test for Al_2_Pt were observed (Figure 3 c). Only a standard (short‐term) EC experiment was performed for elemental Pt, aiming to compare the electrochemical activity of Al_2_Pt vs. Pt.

**Figure 4:**
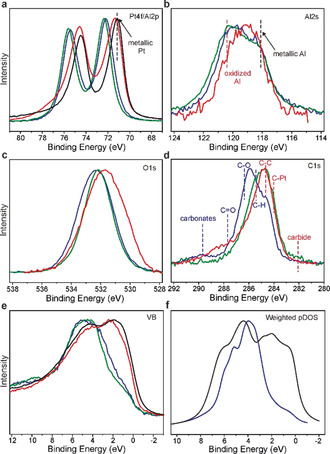
X‐ray photoelectron spectroscopy of Al_2_Pt in different states (as‐synthesized (blue), sputtered (green), and after the OER experiment (red)) compared to elemental Pt (black). The results are represented via a) Pt 4f, b) Al 2s, c) O 1s, d) C 1s XPS core levels, as well as e) experimental valence bands and f) sum of the calculated weighted and broadened partial DOS for Al_2_Pt. The intensities of all XP spectra have been normalized.

To investigate the composition and the electronic state of the surface atoms, the material was characterized by X‐ray photoelectron spectroscopy (XPS) in three states: 1) as‐synthesized, 2) after Ar sputtering accompanied by gentle heating, and 3) after OER reaction (Figure 4, Table 1). The near‐surface region of the as‐synthesized material contains a mixture of Al_2_Pt with some aluminium oxide and carbon caused by polishing of the material. After sputtering, a significant portion of the carbon is removed, whereas there is only little change in the oxygen content. This agrees with the Al 2s spectrum, suggesting that even after sputtering most of the aluminium is oxidized, representing the expected alumina overlayer. The ratio is roughly 2/3 oxidized and 1/3 metallic Al. The Al/Pt ratio is very large (Table 1), but after correcting for the aluminium oxide, it drops to 2.1:1, which is essentially the stoichiometry of the compound Al_2_Pt. Using the Al 2s fit (2/3 oxidized component), the calculated Al/O ratio for oxidized Al is 1:1.45, very close to the stoichiometric Al_2_O_3_. The Pt 4f core level shift is +1.0 eV for the as‐synthesized sample and +1.2 eV for the sputtered sample compared to metallic Pt, revealing the strongly modified electronic state of Pt in the intermetallic compound. Density functional theory (DFT) calculations (cf. Section 1.4 in SI) predict a shift of 1.47 eV, which is close to the observed value. The valence band features—a strongly reduced density of states at the Fermi level and the narrowing of the Pt d band—indicate the intended modification of Pt out of its collective metallic electronic structure into an atom‐like state (Figure 4 f). Although DFT predicts that the d band is composed of at least three peaks (Figure S3), which are not resolved in the experiment, the shift of the d band as well as its narrowing are quite well reproduced.

**Table 1:** Molar ratios of Al_2_Pt‐based material in the near‐surface region (XPS data).

State	Al/Pt	O/(Pt+Al)	C/(Pt+Al)
as‐synthesized	5.0	1.11	1.21
sputtered	6.2	0.83	0.37
after OER	0.47	1.19	1.32

XPS analysis of the sample after a standard OER experiment (LSV followed by 2 h of CP operation and LSV measurement afterwards) clearly shows that the sample undergoes Al leaching under OER conditions. The Al/Pt ratio (0.47) is far lower than in the as‐prepared state, and the Al is present in both—metallic and oxidized—states. The main Pt 4f line of this sample is very broad and asymmetric towards high binding energies. This might be due to either a significant Pt^2+^ contribution or the contribution from the bulk Al_2_Pt phase. The sample contains in addition Pt^4+^ species and Cl impurities. The former is due to some oxidation of Pt during the experiment (cf. long‐term experiment below). The latter may arise from the electrolyte.

**Figure 5:**
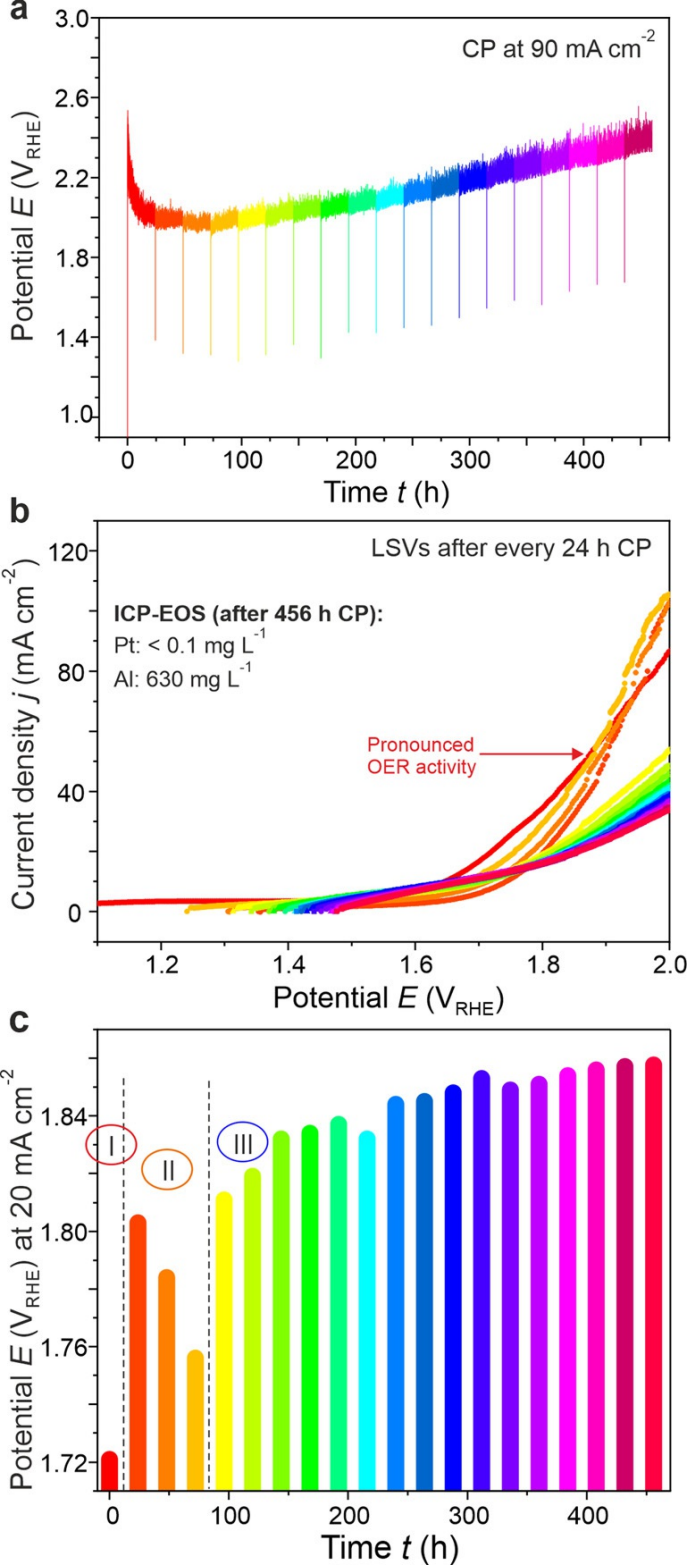
Long‐term stability of Al_2_Pt under OER conditions: a) chronopotentiometry at a current density of 90 mA cm^−2^ for 456 h, along with b) series of LSV curves, recorded at 24 h intervals of CP. c) Variation of potential, necessary to reach 20 mA cm^−2^, as a function of CP operation time. The colors denote 24 h intervals during the long‐term experiment in 0.1 m HClO_4_.

In order to assess the stability of the system^40^, ^41^, ^42^ after transformation into the activated form, we carried out a long‐term test addressing the resistance of the system against continuous de‐alloying.^43^, ^44^, ^45^, ^46^, ^47^ The long‐term CP measurement (456 h at 90 mA cm^−2^) was accompanied by LSV records every 24 h for additional monitoring of the activity response (Figure 5). Chronopotentiometry clearly reveals the activation of the material during initial 96 h of CP, followed by monotonic reduction of the catalytic activity for the remaining time of the experiment (Figure 5 a). The LSV curves reflect these results (Figure 5 b).

The superior activity, obtained after first 24 h of operation and lasting for another 72 h, is followed by a shape change indicative of enhanced transport limitations. Transformation of the initially gel‐like active layer^48^ into a crystalline Pt compound^49^, ^50^ and/or a re‐precipitated aluminum oxide surface phase represents a scenario of formation and gradual passivation of an active OER termination layer. It is important that the degradation does not lead to the failure of the active layer over long times and whole complex structure (cf. details below) still possesses sufficient electrical conductivity to warranty electrochemical performance. The activity response during the long‐term stability experiment is summarized in Figure 5 c using 20 mA cm^−2^ as a marker for electrochemical activity. The slowly decreasing activity after 100 h of operation (exceptional duration of stability test in fundamental studies!) at relatively high current density (90 mA cm^−2^ instead of commonly used 10 mA cm^−2^) is mainly because of reduced conductivity of the surface layer due to PtO_*x*_ formation and re‐precipitation of aluminum oxide. The ex situ elemental analysis of the working electrolyte after long‐term experiment detects a significant amount of aluminum (630 mg dm^−3^), whereas no platinum was lost, in agreement with earlier observations.^27^, ^51^ These findings collectively point toward a gradual transformation of the initial intermetallic compound into a composite material, as intended by our strategy.

**Figure 6:**
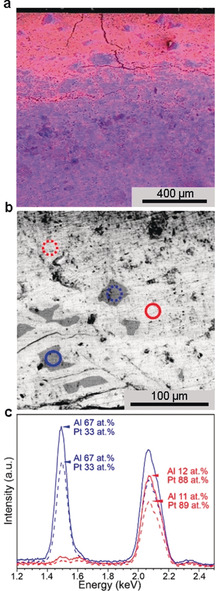
SEM characterization of Al_2_Pt after long‐term electrochemical experiment (see Figure 5). a) EDX spectrum image of the top 1.2 mm of the sample (cross section view; Al‐rich domains in blue, Pt‐enriched grains in red). The upper part in the image corresponds to the area in contact with the electrolyte. b) Back‐scattered electron (BSE) image with areas corresponding to the energy‐dispersive X‐ray spectra (EDX) shown in (c). For elemental mapping in samples after long‐term EC, see Figure S7.

To gain insight into the morphological aspects of the self‐organized transformation, scanning electron microscopy (SEM) and energy‐dispersive X‐ray (EDX) spectroscopy of mechanical cross section of the used electrode were carried out (Figure 6).

The complex structure of the specimen from its surface to the bulk is shown in Figures 6 a and S4. The surface of the sample is characterized by significant number of cracks, extending into the sample. They were formed during the densification of Al_2_Pt specimen via spark plasma sintering (SPS, Supporting Information S1.1). Such cracks provide an access for the electrolyte into the bulk material and serve as a route for aluminum leaching. The depth of Al leaching is visible where a phase of bright intensity is observed to mix with a complex and dense structure of dark contrast grains at about 400 μm. To corroborate the leaching, the elemental compositions of the two distinguished phases were determined by EDX spectroscopy (Figure 6 c). Accordingly, the dark areas, that is, grains, in the BSE image (Figure 6 b) have an average composition of 67±3 at. % Al and 33±1 at. % Pt and the bright areas correspond to Al‐depleted material (Al 12±1 at. % and Pt 88±3 at. %). The leaching takes place either at the surface of the specimen or around the cracks and voids in the material (Figures S5 and S6). No formation of nanoparticle‐like platinum precipitation is observed within the layer.

Summarizing the electrochemical, XPS, and SEM results, the application of anodic potentials to Al_2_Pt leads to noticeable leaching of aluminum with formation of metallic Pt (which tends to be oxidized after more than 100 h of CP) on the surface and a composite material of Al‐depleted phases with remnant Al_2_Pt in the bulk.

## Conclusion

The investigation of the intermetallic compound Al_2_Pt as an electrocatalyst for OER clearly shows that:

1. The material undergoes restructuring during the first 100 h of OER operation in the near‐surface region.
2. The restructured volume is a composite of a platinum‐rich phase (e.g., solid‐solution of Al in Pt), some oxidized Pt, and aluminum oxide as a mineral spacer.
3. The bulk material retains its structural and compositional integrity after 456 h of anodic operation at 90 mA cm^−2^.
4. Reduced electrical conductivity due to the formation of oxide products at the surface (besides the active gel layer that is fully operational after 100 h of OER) is considered to be responsible for the activity loss during long‐term operation.

These findings support the viability of our strategy. It is essential to begin with an intermetallic compound in order to provide the basic bulk stability of the system. It is further relevant to use a higher concentration of the main group element to modify the electronic structure of platinum. This modification enables its conversion into a partly oxidized hydrated gel phase that optimally reacts with water in a porous structure. It is also important to prevent the crystallization of the gel by intermixing it with a mineral spacer (hydrated alumina) that must not be dense as otherwise the diffusion of reactants and products would be inhibited. This spacer, in addition, functions as a passivation layer to kinetically inhibit the continual dissolution of the non‐noble component in the surface phase. The system is active in electrocatalysis as it represents a material in a frustrated transition involving its oxidative decomposition. These functions were considered in the selection of the starting compound. In combination with the known activity of Pt and Pt‐based intermetallic compounds in the hydrogen evolution reaction (HER),^52^, ^53^ this opens a new strategy in the development of bifunctional electrocatalysts. Thin‐film technologies and the design of a (less costly) bulk phase as a support are avenues for further developing the strategy. Other intermetallic compounds featuring amphoteric non‐noble metals in large fractions are also possible candidates for expanding the material basis of our concept.

## Conflict of interest

The authors declare no conflict of interest.

## Supporting information

As a service to our authors and readers, this journal provides supporting information supplied by the authors. Such materials are peer reviewed and may be re‐organized for online delivery, but are not copy‐edited or typeset. Technical support issues arising from supporting information (other than missing files) should be addressed to the authors.

Supplementary
