# Supplementary material for: Al2Pt for Oxygen Evolution in Water Splitting: A Strategy for Creating Multifunctionality in Electrocatalysis
Source: Angew Chem Int Ed Engl. 2020 Jul 10;59(38):16770–6. doi: 10.1002/anie.202005445 (PMC7539922; doi:10.1002/anie.202005445)
Supplement: Supplementary file 1 — Supplementary [file ANIE-59-16770-s001.pdf]

Angewandte Chemie
Eine Zeitschrift der Gesellschaft Deutscher Chemiker

Supporting Information

Al_2Pt for Oxygen Evolution in Water Splitting: A Strategy for Creating Multifunctionality in Electrocatalysis

Iryna Antonyshyn, Ana M. Barrios Jiménez, Olga Sichevych, Ulrich Burkhardt, Igor Veremchuk, Marcus Schmidt, Alim Ormeci, Ioannis Spanos, Andrey Tarasov, Detre Teschner, Gerardo Algara-Siller, Robert Schlögl, and Yuri Grin**

anie_202005445_sm_miscellaneous_information.pdf

Supporting information

S1. Materials and methods

S1.1. Sample preparation

Platinum granules (Chempur, 99.99 %, 1-6 mm) and pieces of aluminum rod (Alfa Aesar, 99.9965 %) were weighed in atomic Al : Pt ratio equal to 32 : 68. The constituent components were arc melted at least two times with mass losses not exceeding 0.2 %. Obtained ingots were placed into alumina crucibles and sealed under argon into tantalum containers. For oxidation protection at high temperatures, tantalum containers were inserted into quartz glass ampoules and sealed under vacuum. The samples were annealed in a resistance furnace at 1000 ^{o}C for 670 hours with subsequent quenching of quartz ampoules in ice water.

Material discs (necessary for electrochemical experiments, diameter - 8 mm, height - 3–4 mm) were manufactured via Spark Plasma Sintering (**SPS**) technique. The ground powder (agate mortar, argon-filled glovebox) was filled into SPS press-form, which was exposed to the heating by pulsed direct electrical current with low voltage (T_{max} = 1000 ^{o}C at 80 MPa for 10 min).

S1.2. Characterization

The synthesized samples were characterized via powder X-ray diffraction (**PXRD**) using Huber Imaging Plate Guinier Camera G670 (Cu$K\alpha_1$ radiation, λ = 1.54059 Å, LaB_6 with a = 4.1569 Å as internal standard). Quantitative phase analysis of synthesized samples was carried out via comparison of the experimental patterns with the theoretically calculated ones (program WinXPow[1]). WinCSD software package[2] was used for the indexing of diffraction patterns and determination of lattice parameters.

Scanning electron microscopy (**SEM**) was used for the examination of sample homogeneity as well as for the precise determination of the composition. The sample was embedded into conductive polymer and polished with SiC papers and diamond powders with different grain sizes (ending with 1/4 µm diamond powder in slurry). Initially, optical microscopy (Axioplan 2, Zeiss) with bright-field, dark-field, polarized light and differential interference contrast was carried out. Afterwards, elemental analysis of metallographic cross sections was performed with JEOL 7800F with an attached EDXS system (Quantax 400, Bruker, Silicon-Drift-Detector (SDD), FEG cathode, acceleration voltage: 0.1 kV–30 kV). For accurate quantitative analysis,

wavelength-dispersive X-ray spectroscopy (**WDXS**) as an option of the electron microprobe Cameca SX100 (tungsten cathode, acceleration voltage: 1 kV–30 kV) was applied. X-ray intensities were measured at 20 kV using elemental Al (100 %) and Pt (99.999 %) as reference probes for the intensities of Al K*α* and Pt L*α* lines. The PAP matrix correction mode[3] was used for chemical composition calculations.

For the cross-section characterization, the sample was cut in half with diamond saw and polished down with 1 µm SiC paper. The characterization of the cross-section was made in a SEM (10 kV) Hitachi S4800 with SE and BSE (YAGBSE) detectors and Bruker EDX spectrometer (Xflash 6|30).

Differential scanning calorimetry (**DSC**) under argon atmosphere was applied for clarifying the Al_2Pt formation route (Netzsch DSC 404C Pegasus, Al_2O_3 crucible with cap, T_{max} = 1600 °C, heating/cooling rate of 20 °C min^{-1}).

X-ray photoelectron spectroscopy (**XPS**) was used to evaluate the sample composition in the near-surface region as well as to assess the chemical state of the contributing phases/species in as-synthesized samples and after electrochemical experiments. Spectra were recorded at room temperature, using non-monochromatized Al*Kα* excitation (1486.6 eV) and a hemispherical analyzer (Phoibos 150, SPECS). The binding energy scale was calibrated by the standard Au $4f_{7/2}$ and Cu $2p_{3/2}$ procedure. To calculate the elemental composition, theoretical cross sections from *Ref.* 4 were used.

S1.3. Electrochemical measurements

The electrochemical performance of Al_2Pt was investigated in a combined electrochemical flow cell (EFC)/inductively coupled plasma-optical emission spectrometry (ICP-OES) setup. The setup design as well as details on the benchmarking protocol used for the corresponding electrochemical performance evaluation are described elsewhere.[5] A coil-shaped platinized platinum wire (PT-5W, 125 µm diameter, 99.99 %, Science Products GmbH), placed along the flow channel following the electrolyte outlet flow, was used as the counter electrode (CE), while the reference electrodes (RE) (SCE, CH Instruments Inc., CHI150, reference potential +241 mV vs. NHE) was inserted perpendicular to the electrolyte inlet channel to avoid possible Pt corrosion and re-deposition on the WE during the electrochemical testing. The potentiostat used for the electrochemical measurements is a Bio-Logic SP-150, while the EC-Lab software was used to record the obtained data. The resulting electrolyte stream is continuously fed into the ICP-OES (Spectroblue EOP, Ametek)

by means of a peristaltic pump at a flow rate of 0.86 ml/min, through a quartz nebulizer operating at nebulizer gas flow rates of 0.85 L min^{-1} (Ar, purity 99.999 %). All stability tests have been performed in chronopotentiometric mode, by applying constant 10 mA cm^{-2}.

Static electrochemical 3-electrode cell is designed for electrochemical experiments with intermetallic compounds and was used for long-term stability experiment. The pellet of Al_2Pt sample is inserted into the cell from the bottom and tightly fixed to gold electrode which is clamped to measuring potentiostat (SP-300, Bio Logic). Such construction and position of the working electrode in the bottom part of electrochemical cell allow free escape of the gas bubbles during the electrochemical operation. Pt coil (PINE, 99.99 %, Ø = 0.5 mm) and single junction calomel (Hg/Hg_2Cl_2) electrode (PINE, 4 M KCl, reference potential +241 mV vs NHE) were used as counter and reference electrodes, respectively. Aiming at oxygen removal, the electrolyte solution was bubbled with argon flow (Messer, 5.0) for at least 30 min before each experiment. The reference electrode was calibrated against reversible hydrogen electrode (HydroFlex, Gaskatel GmbH) in the same electrolyte. Before the experiment, the working electrode (SPS-manufactured pellet) was polished using SiC papers and diamond powders with different grain sizes implemented into semi-automatic polishing machine (EcoMet250Pro, Buehler). The polished sample was inserted into the Ar-saturated electrolyte, followed by potential cycling between 0.05 and 1.0 V_{RHE} with a scan rate of 50 mV s^{-1} for 50 cycles. To determine OER activity, linear sweep voltammetry (LSV) was performed in potential range from open circuit potential up to 2.0 V_{RHE} with a scan rate of 5 mV s^{-1}. Current density of 10 mA cm^{-2} was chosen for comparison of the data because it is a benchmarking value for fundamental studies and testing of electrocatalyst materials for OER.[5,6]

Since the initial LSV reached a maximal current density of 90 mA cm^{-2} at 2.0 V_{RHE}, this value was taken for the harsh stability measurement. The stability was thus judged using chronopotentiometry (CP) technique at a current density of 90 mA cm^{-2} for 456 h. To monitor the activity of the sample during the CP measurement, LSVs were recorded every 24 h. In order to release the system, open circuit potential (OCP) was measured for 5 min before and after LSV scan. In total, there were 19 cycles. The depth of material changes after 456 h long-term experiment is 400 μm, which is less than 10 % from the specimen thickness. Due to a good conductivity of Al_2Pt material,

it works as a current collector. From our experiments, the thickness of the pellet may impact only technical characteristics and tightness of the EC cell, but not the measured overpotential of the material.

All measurements were done in freshly prepared 0.1 M $HClO_4$ solution (by dilution of 70 % $HClO_4$ (Aldrich, 99.999 % trace metal basis) in millipore water with resistivity of 18.2 MΩ cm). The potentials are expressed vs RHE potential scale and internal resistance (IR) correction was assumed in all measurements. For current density calculations, the current values were divided by the geometrical surface area of the electrode (0.204 cm^2).

S1.4. Theoretical calculations

Electronic structure calculations were performed for the ordered model Al_2Pt (space group $Fm\bar{3}m$, a = 5.9190 Å) on the fully-relativistic level by using the all-electron, full-potential local orbital method (**FPLO**)[7] and the **Fritz-Haber Institute ab initio molecular simulations method (FHI-aims)**.[8] The local density approximation to the density functional theory as parameterized by Perdew-Wang[9] was employed to account for the exchange-correlation effects. The Brillouin zone was sampled with a mesh of 24×24×24 k points. The electron density (ED) and the electron localizability indicator (ELI) in its ELI-D representation (Y_D)[10,11] were computed employing the interface implemented in the FHI-aims package.[12] The topological analysis of ED and ELI-D was carried out by the program DGrid.[13]

The projected densities of states (DOS) corresponding to the states occupied in the free atom electronic configurations were convoluted by a Lorentzian function with a broadening of 0.5 eV. These were then multiplied by the photoionization cross sections[4] and their sum was compared to the valence band spectrum obtained by XPS.

The core level shifts were calculated using the delta SCF method.[14] Calculations were performed using PBE exchange[15] and correlation potential using the Quantum ESPRESSO package[16] with norm-conserving pseudopotentials with the kinetic energy cutoff of 40 Ry and k-point mesh of 10×10×10 and a Marzari-Vanderbilt smearing[17] with the parameter of 0.02 Ry.

S2. The phase Al_2Pt

According to our studies, the Al_2Pt phase possesses small homogeneity region with compositions deviating slightly from stoichiometry 2:1. There is ongoing project dedicated to the structural features and nature of such compositional changes of Al_2Pt, which will be the topic of a separate publication. Concerning the sensitivity of catalytic properties (particularly OER activity) towards the composition, at least initial OER activity for all compositions is the same within the sensitivity range of applied methods.

S3. Supporting figures

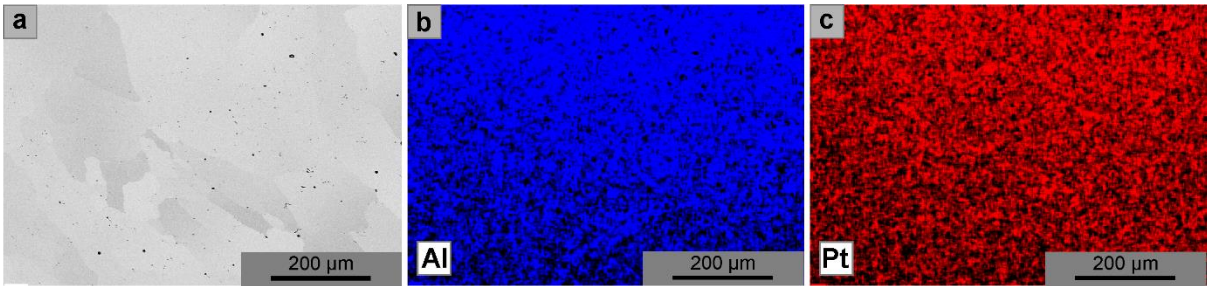

Figure S1. Microstructure of as-synthesized Al_2Pt: (a) BSE image (30 kV, material contrast), (b) Al and (c) Pt elemental mapping for this region.

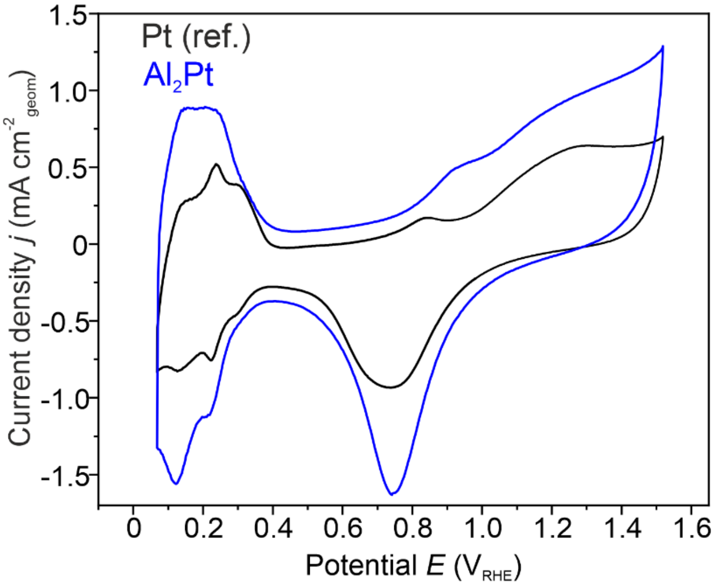

Figure S2. Cyclic voltammetry on Pt (*black*) and Al_2Pt (*blue*): 0.1 M $HClO_4$, 0.05-1.5 V_{RHE}, scan rate 150 mV s^{-1}, 50 cycles.

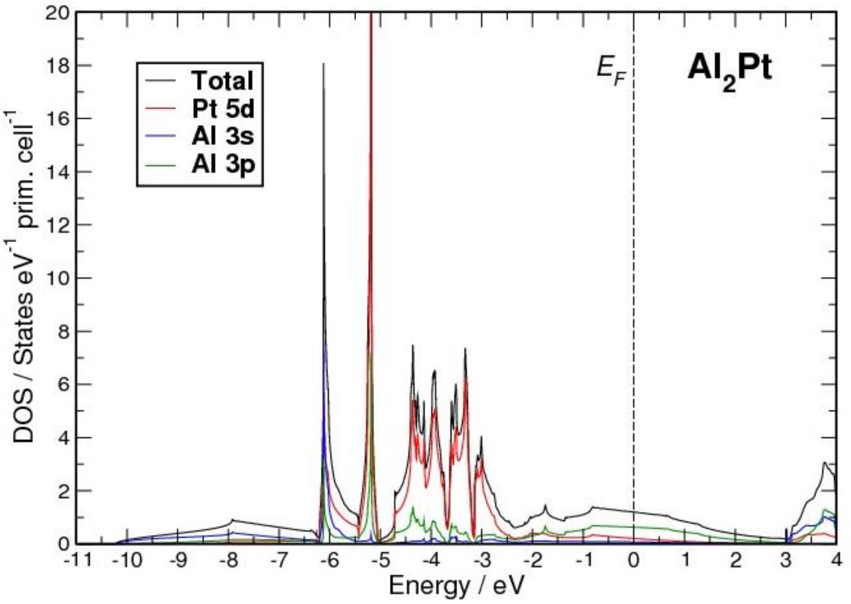

Figure S3. The electronic DOS computed for Al_2Pt using the idealized structure and fully-relativistic treatment.

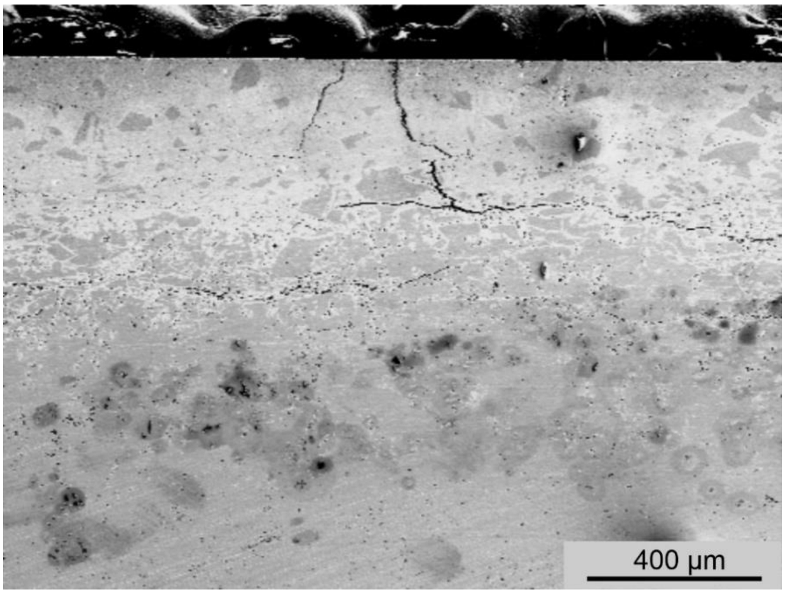

Figure S4. SE image from the spectrum image of Figure 6a: the contrast differences follows the same Al- rich and Pt- rich areas as in Figure 6a in the main manuscript.

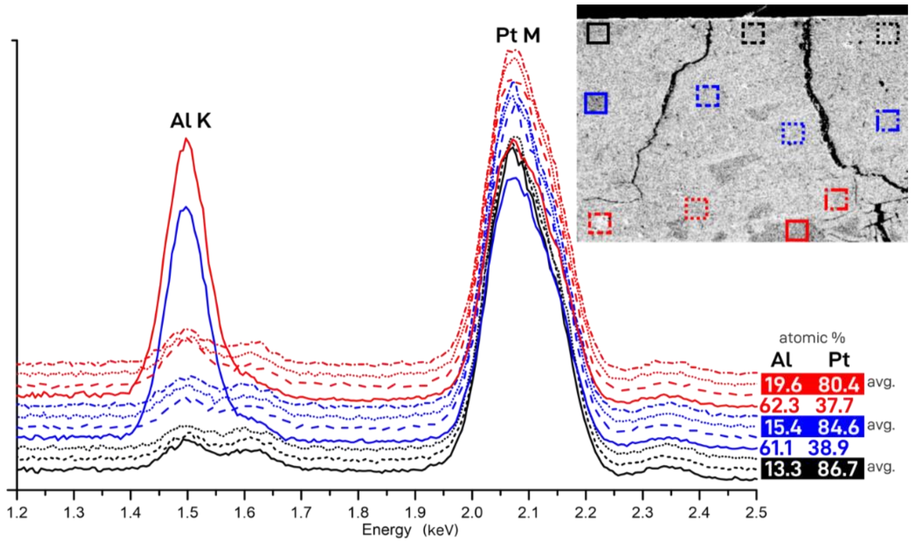

Figure S5. EDX spectra for Al_2Pt material after long-term EC experiment: surface regions (*black*), regions at 100 µm (*blue*) and 200 µm (*red*) into the bulk. Solid lines represent areas with compositions close to Al_2Pt stoichiometry (also visible in BSE contrast). The numbers in white are average composition (in at.%) of three regions marked by dashed lines.

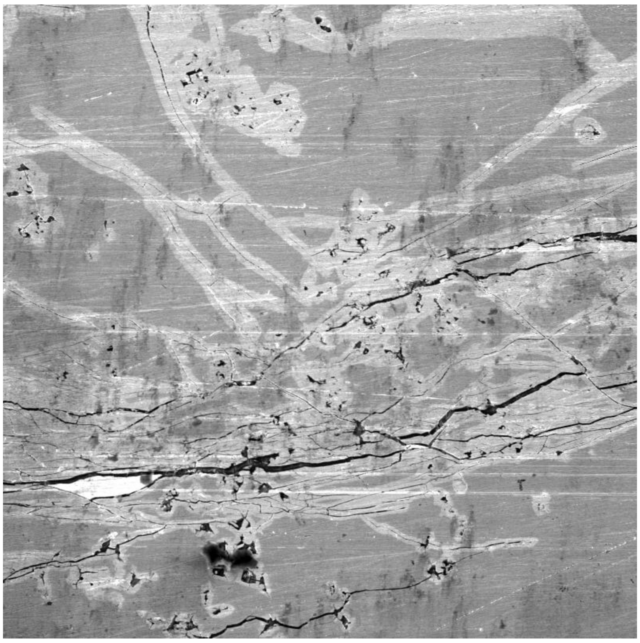

Figure S6. Cracks in the microstructure of Al_2Pt material after long-term EC experiment (456 h of CP at 90 mA cm^{-2}): areas of Al-depleted phase (bright contrast) around cracks and voids (image width 50 µm).

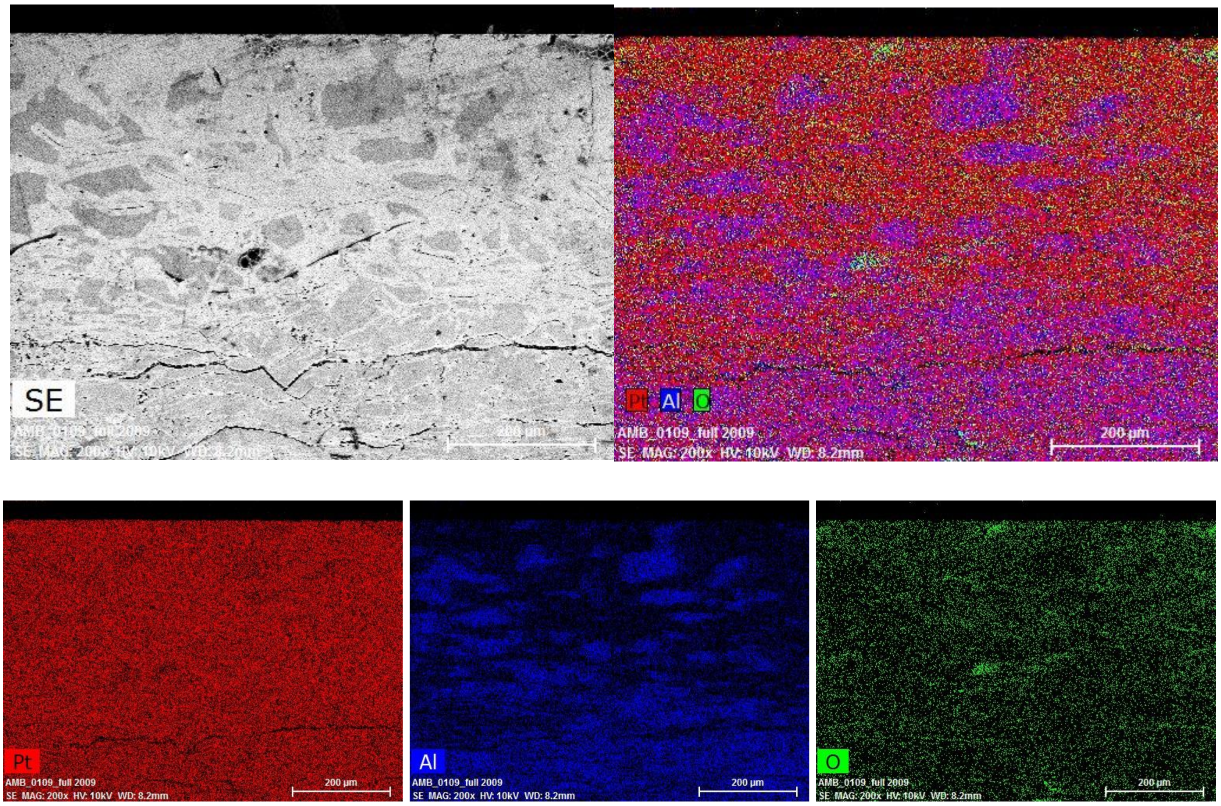

Figure S7. Elemental mapping in the sample after long-term EC experiment: while the localization of Al and Pt can be clearly ascribed to the different regions of the microstructure, the distribution of oxygen cannot be conclusive.

References

[1] *WinXPow, version 2.25*, STOE and Cie GmbH, Darmstadt (Germany), **2009**.

[2] L. Akselrud, Yu. Grin, *J. Appl. Crystallogr.* **2014**, *47*, 803–805.

[3] J. L. Pouchou, F. Pichoir, *Rech. Aerospatiale* **1984**, *3*, 167–192.

[4] J. J. Yeh, I. Lindau, *Atomic Data and Nuclear Data Tables* **1985**, *32*, 1–155.

[5] I. Spanos, A. A. Auer, S. Neugebauer, X. Deng, H. Tüysüz, R. Schlögl, *ACS Catal.* **2017**, *7*, 3768–3778.

[6] C.C.L. McCrory, S. Jung, J.C. Peters, T.F. Jaramillo, *J. Am. Chem. Soc.* **2013**, *135*, 16977–16987.

[7] K. Koepernik, H. Eschrig, *Phys. Rev. B* **1999**, *59*, 1743–1757.

[8] V. Blum, R. Gehrke, F. Hanke, P. Havu, V. Havu, X. Ren, K. Reuter, M. Scheffler, *Comput. Phys. Commun.* **2009**, *180*, 2175–2196.

[9] J. P. Perdew, Y. Wang, *Phys. Rev.* B **1992,** *45*, 13244–13249.

[10] M. Kohout, *Faraday Discuss.* **2007**, *135*, 43–54.

[11] F. R. Wagner, V. Bezugly, M. Kohout, Yu. Grin, *Chem. Eur. J.* **2007**, *13*, 5724–5741.

[12] S. Alarcón Villaseca, A. Ormeci, S. V. Levchenko, R. Schlögl, Y. Grin, M. Armbrüster, *Chem. Phys. Chem.* **2017**, *18*, 334–337.

[13] M. Kohout, *Program DGRID, version 4.6*, Radebeul (Germany), **2011**.

[14] E. Pehlke, M. Scheffler, *Phys. Rev. Lett.* **1993**, *71*, 2338–2341.

[15] J. P. Perdew, K. Burke, M. Ernzerhof, *Phys. Rev. Lett.***1996**, *77*, 3865–3868.

[16] P. Giannozzi, S. Baroni, N. Bonini, M. Calandra, R. Car, C. Cavazzoni, D. Ceresoli, G. L. Chiarotti, M. Cococcioni, I. Dabo, A. Dal Corso, S. de Gironcoli, S. Fabris, G. Fratesi, R. Gebauer, U. Gerstmann. C. Gougoussis, A. Kokalj, M. Lazzeri, L. Martin-Samos, N. Marzari, F. Mauri, R. Mazzarello, S. Paolini, A. Pasquarello, L. Paulatto, C. Sbraccia, S. Scandolo, G. Sclauzero, A.P. Seitsonen, A. Smogunov, P. Umari, R. M. Wentzcovitch, *J. Phys. Condens. Matter* **2009**, *21*, 395502.

[17] N. Marzari, D. Vanderbilt, A. De Vita, M. C. Payne, *Phys. Rev. Lett.* **1999**, *82*, 3296–3299.
